# Use of Piezoelectric Devices in Closed Structural Rhinoplasty

**DOI:** 10.1093/asjof/ojag021

**Published:** 2026-02-03

**Authors:** Sina Kaderi, Can Ekinci

## Abstract

**Background:**

Closed rhinoplasty techniques are gaining popularity once again as more and more patients demand them. The use of piezoelectric devices (PEDs) is also another popular practice in rhinoplasty surgeries recently to reduce postoperative edema and bruising.

**Objectives:**

The aim of this study was to propose a new technique in which all osteotomies in closed rhinoplasty surgeries can be performed using PEDs.

**Methods:**

A comparison was made between 274 patients who underwent closed piezo (CloPi) rhinoplasty and 120 patients who underwent closed rhinoplasty using conventional osteotomy. In the CloPi technique, all osteotomies, including lateral and transverse osteotomies, were performed with PEDs using straight and 90° curved tips. Preoperative and postoperative results obtained with the Rhinoplasty Outcome Evaluation (ROE) questionnaire were compared to evaluate aesthetic and functional results.

**Results:**

In the CloPi group, the mean age was 30.8 years (range, 18-60 years) and the mean follow-up period was 19.6 months, whereas in the control group, the mean age was 33.5 years (range, 18-61 years) and the mean follow-up period was 29.3 months. Significantly better results were obtained in the ROE questionnaire compared with preoperative values in both groups (*P* < .001). When comparing the postoperative–preoperative change in ROE scores between the 2 groups, the improvement observed in the CloPi cohort was significantly greater for all items (*P* < .001), except for Question 2—assessing the ability to breathe—where no significant difference was detected (*P* = .231).

**Conclusions:**

CloPi rhinoplasty is an easy and reliable technique in which all osteotomies can be performed with PEDs. Successful surgical results can be achieved with wide surgical dissection.

**Level of Evidence:**

3 (Therapeutic) 
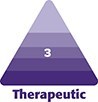

Rhinoplasty and septorhinoplasty procedures are among the most frequently performed aesthetic plastic surgery procedures worldwide.^1^ Although several different techniques are described in the literature, they can be basically divided into 2 main categories: open rhinoplasty and closed rhinoplasty. On the other hand, the choice of technique should be made according to both the patient and the experience of the surgeon.^2^

Although there are different techniques, steps like hump removal, osteotomy, and tip plasty are often performed routinely. Although these steps are similar in different rhinoplasty techniques, various approaches can be used for each step, including osteotomies.^3,4^ These various approaches can be roughly divided into 3 groups according to the tools used: conventional osteotomes, power microsaws or piezoelectric devices (PEDs). The main morbidities seen in rhinoplasty procedures, such as fragmented fractures in the nasal bones, damage to the cartilages, and the amount of edema and ecchymosis, vary depending on the preferred instruments.^5^

The use of PEDs is considered a major breakthrough in rhinoplasty operations.^6^ PEDs selectively cut mineralized structures such as bone with micro-vibrations at ultrasonic frequency while protecting the soft tissue underneath.^7^ Thus, because the damage to soft tissues is drastically reduced, swelling and bruising will also be significantly diminished while accelerating the recovery time. Furthermore, utilization of conventional osteotomes can cause unstable fractures and limits soft-tissue dissection of the bone pyramid, hoping to ensure the stability with soft-tissue envelope.^8^

Although the use of PEDs has become widespread, their utilization in closed rhinoplasty cases, especially in the lateral osteotomy of long nasal bones and transverse osteotomy, is limited because of the narrow surgical area.^1^ To the best of our knowledge, there is only one publication in which all osteotomies were performed with PEDs in closed rhinoplasty, and in this publication, PED was used in preservation rhinoplasty cases.^8^ In closed rhinoplasty with the use of PEDs, hand saws or 2 mm osteotomes are generally used to perform transverse osteotomies.^9^

In this study, we aim to present our novel technique using PEDs within a wide dissection area in order to perform all the osteotomies in closed structural rhinoplasty cases. We present our 3-year experience with the technique and our functional and aesthetic results and compare these results with the results of the control group, which used conventional osteotomies in closed rhinoplasty.

## METHODS

The study was approved by the University Noninterventional Clinical Research Ethics Committee (March 27, 2025 date and 23 number). Medical and personal records of 274 patients operated between May 2021 and May 2024 using closed piezo (CloPi) structural rhinoplasty technique and 120 patients operated between September 2019 and May 2024 using conventional osteotomy in closed rhinoplasty technique were retrospectively compared. Informed consents were obtained from all the patients included in the study, and additional written informed consents for patient information and images to be published were provided by the patients for whom identifying information is included in this article. All of the patients were primary rhinoplasty cases and operated on using either CloPi structural rhinoplasty technique or closed rhinoplasty with conventional osteotomies by a single surgeon at private practice.

Inclusion criteria required at least 3 months of follow-up period, patients being older than 18 years, preoperative and postoperative photographic documentation, and completion of a Rhinoplasty Outcome Evaluation (ROE) questionnaire (Table 1) which is an easy-to-use questionnaire allowing comprehensive assessment of patient satisfaction.^10,11^ The ROE questionnaire was routinely completed by patients at the third, sixth, and 12th postoperative months. For the purposes of this study, only the most recent questionnaire scores submitted by each patient were included in the analysis, and statistical evaluations were performed accordingly. Secondary rhinoplasty cases were not included in the study. Preoperative complaints, surgical indications and desires, intraoperative findings, postoperative complications and complaints like infection, epistaxis, loss of the structural support, tip deprojection, and requests for revision surgery were evaluated in detail.

**Table 1.** Rhinoplasty Outcomes Evaluation (ROE) Questionnaire^11^

Point score	0	1	2	3	4
Q1. How well do you like the appearance of your nose?	Not at all	Somewhat	Moderately	Very much	Completely
Q2. How well are you able to breathe through your nose?	Not at all	Somewhat	Moderately	Very much	Completely
Q3. How much do you feel your friends and loved ones like your nose?	Not at all	Somewhat	Moderately	Very much	Completely
Q4. Do you think your current nasal appearance limits your social or professional activities?	Always	Usually	Sometimes	Rarely	Never
Q5. How confident are you that your nasal appearance is the best that it can be?	Not at all	Somewhat	Moderately	Very much	Completely
Q6. Would you like to surgically alter the appearance or function of your nose?	Definitely	Most likely	Possibly	Probably not	No

Q, question.

### Surgical Techniques

**Figure 1.**
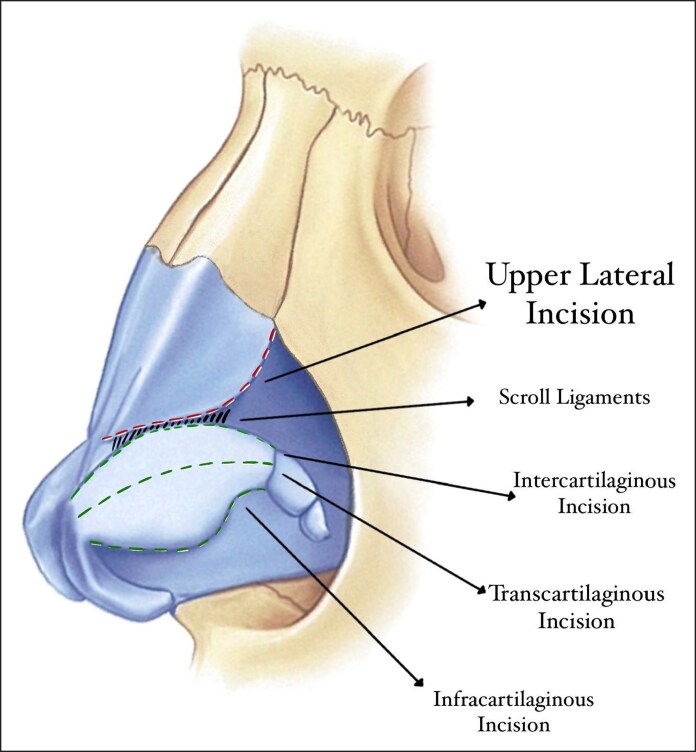
Illustration of the differences between upper lateral incision and other incisions.

A total of 274 patients were operated by using CloPi structural rhinoplasty technique, whereas 120 patients in the control group were operated the by closed rhinoplasty approach with conventional osteotomies. All patients underwent surgery under standard general anesthesia conditions. After nasal cleaning, in the CloPi technique, the caudal border of the upper lateral cartilage (ULC) which is more cephalically positioned compared with the intercartilaginous incision is marked and incised with a scalpel (Video 1). We named this incision as the upper lateral incision because it is more cephalic compared with the intercartilaginous incision and is located at the caudal border of the ULCs (Figure 1, Video 2). This incision was designated at the ULC border, unlike the incisions used in the literature, which were designated according to the lower lateral cartilages. Another advantage of using the upper lateral incision is that the scroll ligaments can be easily preserved because the incision is more cephalic compared with the scroll ligaments. After marking and incising the upper lateral incision, 10 mL of 1% lidocaine hydrochloride and 1:100,000 epinephrine were injected into all dissection and osteotomy lines from the upper lateral incision.

If necessary, radiofrequency ablation and lateralization of the turbinates are performed. A transfixion incision is performed at the most cephalic portion possible. The reason for this is to leave sufficient distance from the marginal incision made during tip plasty and to preserve the circulation of the flap between the transfixion and the marginal incisions. The upper lateral incision and the transfixion incision are joined. It is important to choose an upper lateral incision, as dissection through this incision helps the ULC to come out easily, as in the cartilage delivery technique, and facilitates access to the nasal bone and dorsum.^12^ Supraperichondrial dissection is performed with Colorado needle electrocautery, starting from the dorsocaudal aspect of the septum and progressing toward the ULCs. It is important to begin the dissection supraperichondrially because the next dissection plane is the subperiosteal plane, which is the continuation of this plane.^13^ A wide area was dissected throughout the entire nasal bone extending to the maxilla. This dissection area was then advanced to the cranial dorsum (Supplemental Figure 1). The dissection of the contralateral nasal side is performed identically, and before septum dissection, adrenaline-impregnated nose pads are placed in the dissection areas.

The ULCs are then separated from the septum, and subperichondrial septum dissection begins caudally and progresses cranially and posteriorly to reveal the maxillary crest and septal spur. A cartilage graft is taken from the base of the deviated septum. Then, using a PED straight insert tip, the maxillary crest and any septal spur are cut and excised (Video 3). Afterwards, adrenaline-impregnated nose pads are placed inside the nasal cavity, and the pads on the dorsum are removed. The dome-defining point is determined, and caudal septum excision is performed accordingly.

**Figure 2.**
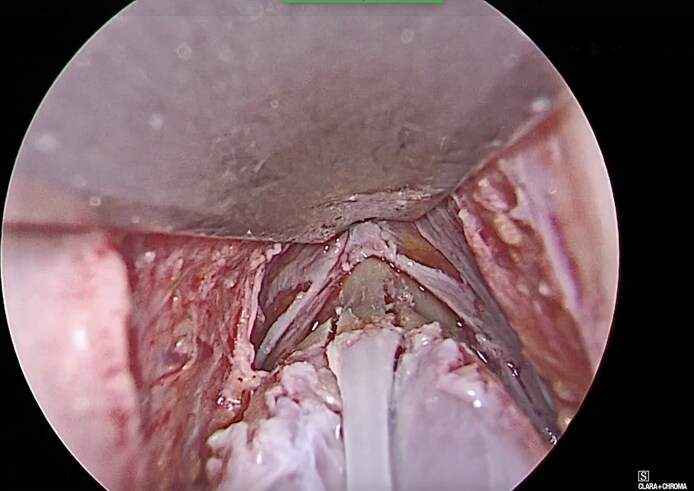
Endoscopic intraoperative photograph showing the paramedian osteotomy in CloPi surgery.

**Figure 3.**
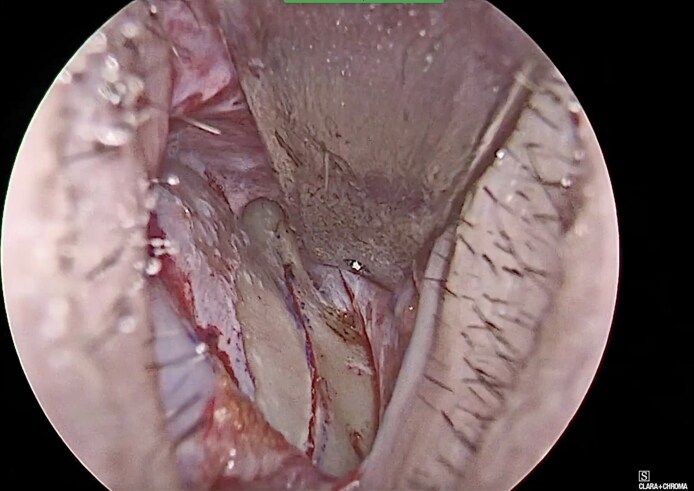
Endoscopic intraoperative photograph showing low-to-low lateral osteotomy in CloPi surgery.

The perichondrium of the uppermost and medial part of the ULCs is peeled off and excised by 2 mm so that the ULCs will bend inward toward the septum according to Gibson's law (Video 4).^14^ In order to prevent damage to the ULCs during hump removal, the ULCs are separated from the bony hump in the keystone region and folded inward toward the septum down to the hump removal line. Afterwards, the bony hump is determined and excised with PED. Similarly, the cartilaginous septal hump is identified and excised with a scalpel. Then, paramedian osteotomies (Figure 2) are performed with the PED straight insert tip up to the frontonasal junction. Low-to-low lateral osteotomies (Figure 3) are performed similarly with the PED straight insert tip (Video 5). If the nasal base is wide or there is a deviated nasal bone, a wedge resection is performed by making a double osteotomy on the desired side or on both sides.

**Figure 4.**
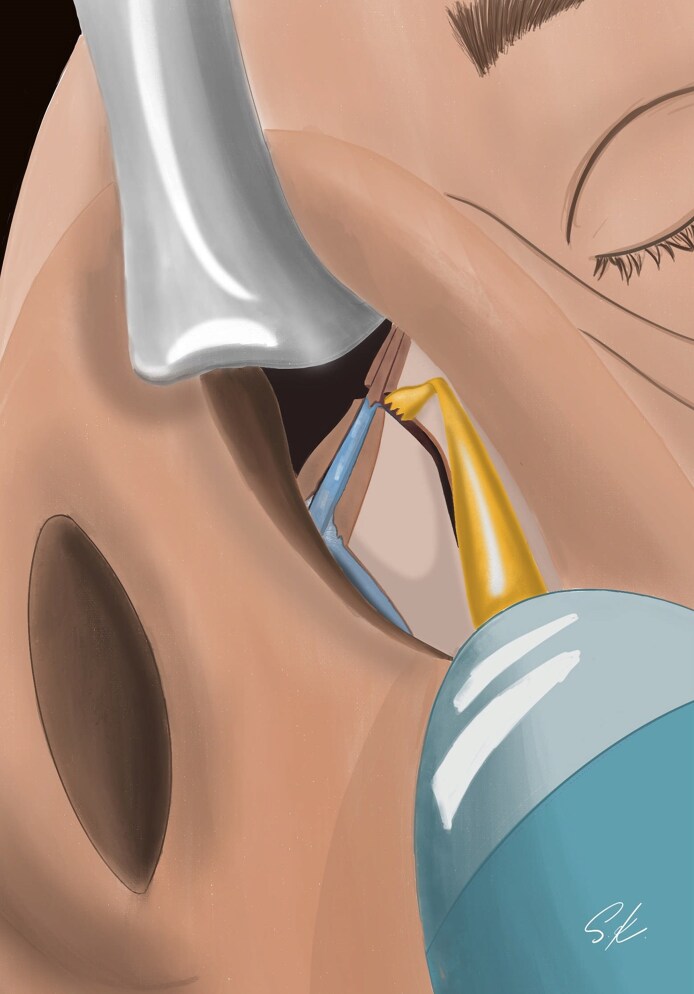
Illustration of the transverse osteotomy with the use of 90°-bent piezoelectric device insert tip.

**Figure 5.**
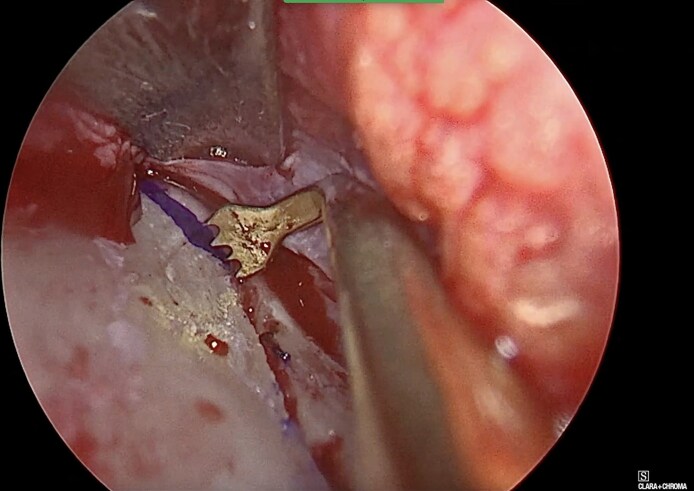
Endoscopic intraoperative photograph showing transverse osteotomy line marking and 90°-bent piezoelectric device insert tip ready for cutting.

Afterwards, transverse osteotomies are performed with 90°-bent PED insert tips (Figures 4, 5) up to the frontonasal junction (Video 6). After all osteotomies are completed, the nasal bones are medialized to close the open roof deformity. ULC fold-in flap or spreader grafts are used to reconstruct the internal nasal valve.^15,16^ Upper lateral and transfixion incisions are closed with 5/0 Vicryl Rapide (Ethicon Inc., Somerville, NJ).

Nasal tip plasty begins with marking the marginal incisions and injecting 2 mL of 1% lidocaine hydrochloride and 1:100,000 epinephrine. After marginal incisions, lower lateral cartilages (LLCs) are exposed by the delivery method.^12^ After determining the new dome, cephalic resection is performed to reduce the lateral crural width if necessary. Although lateral crural reshaping can be performed with different methods such as cephalic excision, sliding alar cartilage flap, or lateral crural turn-in flap, the crucial point is to leave lateral crural width as 4 mm in the dome region and 8 mm in the most cephalic part.^17-19^ Hemitransdomal sutures are placed in the newly defined domes, and the LLCs are exposed through a single nostril by cartilage delivery technique. After the dome-equalizing suture, the columellar strut graft is placed. The medial crural overlap procedure is performed. Then, the columellar strut is sutured to the medial crura, and the newly formed nasal tip is put in its place. All incisions are closed with 5/0 Vicryl Rapide. A plaster cast and intranasal Doyle splint are applied and both are removed in the first week.

In the control group, the closed rhinoplasty technique was performed using routinely described techniques without any special maneuvers.^20^ All osteotomies (paramedian, low-to-low lateral, and transverse osteotomies) were performed with conventional osteotomes. Nasal tip plasty was performed as described above.

### Statistical Analysis

IBM SPSS (IBM Corp., Armonk, NY) for Windows 21 was used in data analysis. The Kolmogorov–Smirnov test was used to assess the normality of the variables. Nonparametric tests were used for group comparisons. The Mann–Whitney *U* test, a nonparametric test, was used for group comparisons. Crosstabs were prepared according to the preoperative and postoperative values obtained according to ROE questionnaire, and the χ^2^ test was used in the analysis. For the presentation of the data, mean ± standard deviation values were used. The significance level was set at *P* < .05.

### Ethical Approval

All procedures followed were in accordance with the ethical standards of the responsible committee on human experimentation (institutional and national) and with the Declaration of Helsinki 1975, as revised in 2013. The study was approved by the University Noninterventional Clinical Research Ethics Committee (March 27, 2025 date and 23 number). Informed consents were obtained from all the patients included in the study. Additional written informed consents for patient information and images to be published were provided by the patients for whom identifying information is included in this article.

## RESULTS

In the CloPi group, patient ages ranged from 18 to 60 years, with a mean age of 30.8 years. The cohort consisted of 104 females and 62 males (Supplemental Table 1). In the control group, patient ages ranged from 18 to 61 years, with a mean age of 33.5 years, and the distribution included 76 females and 44 males (Supplemental Table 2). Follow-up duration ranged from 3 to 38 months (mean: 19.6 months) in the CloPi group and from 3 to 58 months (mean, 29.3 months) in the control group. Both age (*P* = .045) and follow-up duration (*P* < .001) were significantly higher in the control group than in the CloPi group (Table 2). All patients underwent either primary CloPi rhinoplasty or closed rhinoplasty with conventional osteotomies; secondary rhinoplasty cases were not included in the study. Most patients underwent surgery because of aesthetic concerns (50.2%), a few because of breathing problems (6.3%), and some to solve both problems (43.4%).

**Table 2.** Demographic Characteristics of the closed piezo (CloPi) and Control Cohorts with *P*-value Comparisons

Variable	CloPi group (*n* = 274)	Control group (*n* = 120)	*P*-value
Age (years)	30.8 ± 8.7	33.5 ± 10.9	.045
Gender (female, %)	66.8	61.7	.00031
Follow-up duration (months)	19.6 ± 10.2	29.3 ± 16.4	<.001
Revision surgery (%)	2.9	7.5	.699

**Figure 6.**
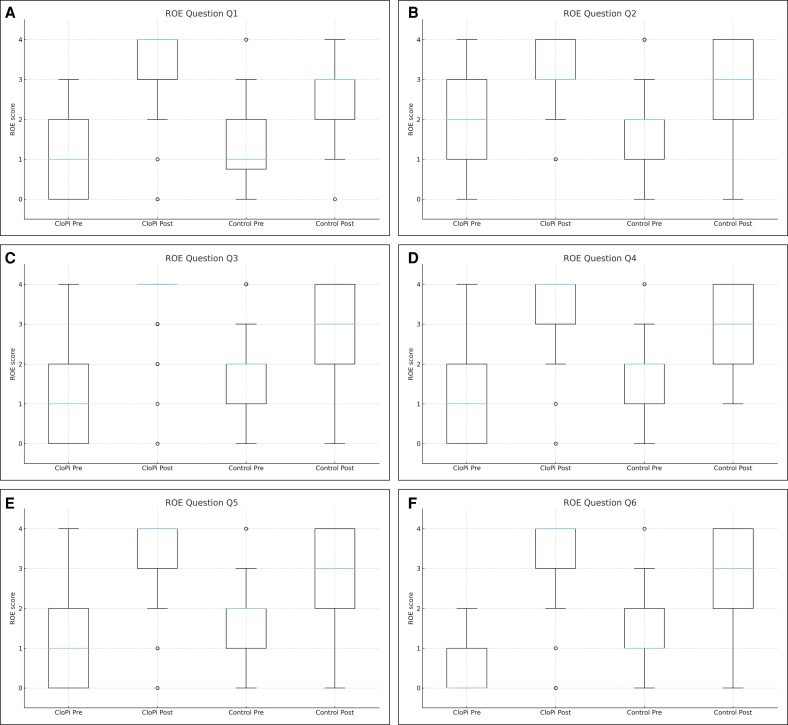
Boxplots of Rhinoplasty Outcomes Evaluation (ROE) item scores in the closed piezo (CloPi) and control groups. (A-F) For each question (Q1-Q6), preoperative and postoperative scores are shown for the CloPi group (left 2 boxes) and the control group (right 2 boxes).

Preoperative and postoperative outcomes were assessed using the ROE questionnaire (Table 1), and statistical analyses were performed to compare the preoperative and postoperative results.^10^ Statistically significant improvements (*P* < .001) were observed in the responses to all questions of ROE questionnaire in both groups (Supplemental Tables 3, 4). When the difference between postoperative and preoperative ROE scores between the 2 groups was examined, it was seen that the improvement observed in the CloPi group was significantly better (*P* < .001) in all questions except the second question (*P* = .231), which specifically assesses functional nasal breathing capacity (Figure 6).

**Figure 7.**
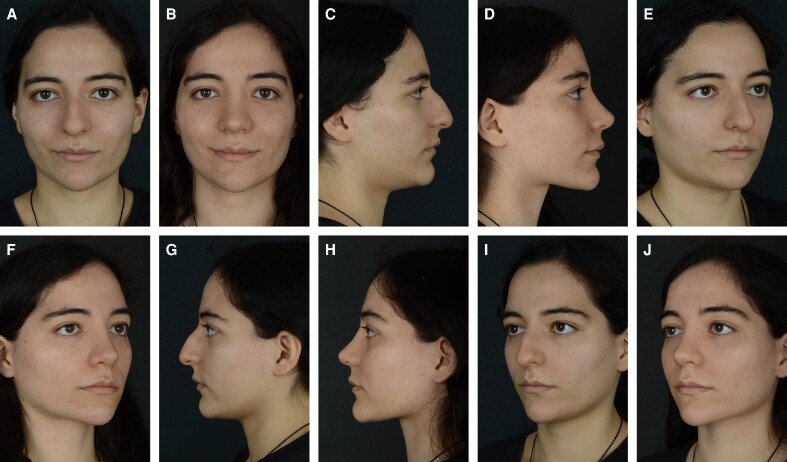
(A, C, E, G, I) Preoperative and (B, D, F, H, J) 12-month postoperative results of a 27-year-old female patient with a dorsal hump and droopy nasal tip who underwent closed piezo (CloPi) rhinoplasty.

**Figure 8.**
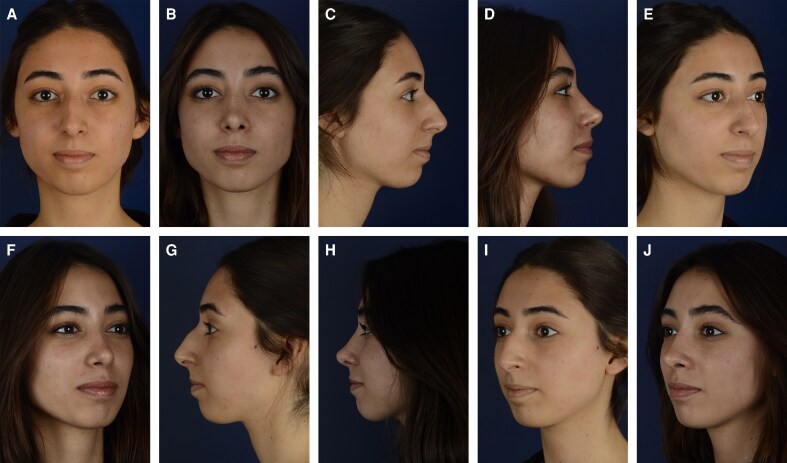
(A, C, E, G I) Preoperative and (B, D, F, H J) 12-month postoperative results of a 23-year-old female patient with a high radix and dorsal hump who underwent closed rhinoplasty with conventional osteotomies.

None of the patients in the CloPi group had any complaints regarding the nasal dorsum. There were only a few patients (*n* = 14) complaining about long-lasting edema after the surgery. Only 8 patients (2.9%) in the CloPi group requested revision surgery during their follow-ups, and the reason for this was nasal tip deprojection in the postoperative period. After surgery, the patients' nasal dorsum and lateral nasal walls were stable, and they had no significant irregularities to complain about. No burns or soft tissue damage associated with PEDs were observed postoperatively (Figure 7). In the control group, revision surgery was requested by 9 patients in total. Two patients (1.7%) sought revision because of nasal wall and dorsum irregularities, whereas 7 patients (5.8%) requested revision because of postoperative tip deprojection that developed during the follow-up period. Although edema was not evaluated using any objective imaging modality, only a small number of patients (*n* = 9) experienced prolonged postoperative edema, similar to the findings in the CloPi group. Overall patient satisfaction in the control group undergoing closed rhinoplasty with conventional osteotomies (Figure 8) remained high.

No major complications or complaints were observed after surgery. Patients in both groups did mention routine postoperative symptoms, including nasal bleeding, congestion, mild pain, and mild facial edema, although all symptoms resolved within a few weeks with routine postoperative care.

## DISCUSSION

Novel rhinoplasty and septorhinoplasty techniques are increasing in number with increasingly rising numbers of patients' demands for the surgery.^1^ In addition to aesthetic results, the comfort provided to the patient while achieving these results is also becoming more and more crucial every day. Perhaps one of the most important improvements in this regard has been the reduction of postoperative edema with the use of PEDs.^8^

Compared with conventional osteotomies, PEDs have been shown to be selective to bone, preserve the underlying and surrounding soft tissues, cause less bruising, swelling, and edema, and have a lower risk of comminuted fractures of the nasal bones.^7^ However, its use is limited, especially in lateral and transverse osteotomies, because the PED tips cannot easily access the surgical field in the closed rhinoplasty surgeries or are difficult to manipulate.^1^

Closed rhinoplasty, or endonasal rhinoplasty, as named back in the old days, is an older and long-established technique compared with open or external rhinoplasty.^21^ However, open rhinoplasty approaches popularized by Goodman in the early 1970s gained tremendous favor because of the ability to easy access to the surgical field and, accurately and clearly diagnose the structural problems through direct imaging, and perform operative techniques easier.^12,21^ Therefore, although more structures are damaged in the open rhinoplasty approach, it is preferred by many because it is easier to perform and learn, and the structures are easier to access, providing more predictable outcomes.^12^

**Figure 9.**
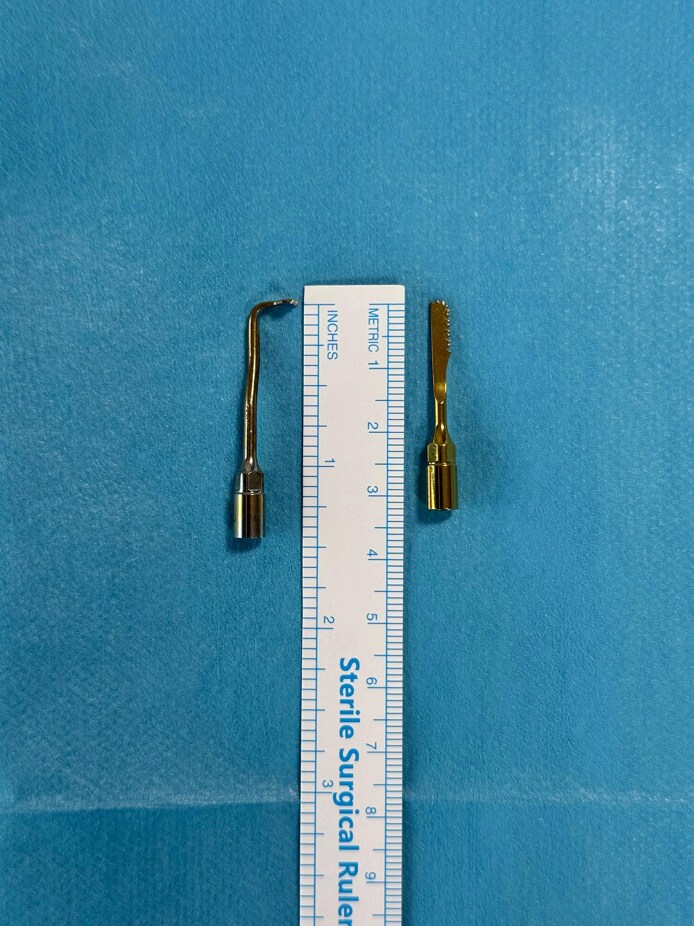
Photograph of the piezoelectric device insert tips used in closed piezo (CloPi); 90°-bent piezoelectric device insert tip and straight piezoelectric device insert tip.

However, recently, interest in closed rhinoplasty, which has been repopularized by Çakır et al, has increased again and has started to become a technique that is more popular among patients.^22^ On the other hand, although different instruments like conventional osteotomes or hand saws can be used for osteotomies in closed rhinoplasty, PEDs cannot be used as much easily as these instruments because of limited access to the surgical field in closed rhinoplasty.^1^ There is only a single study in the literature that we found, performed all osteotomies in closed rhinoplasty using PEDs.^8^ However, CloPi differs from that in that not only it is structural rhinoplasty, but also in that transverse osteotomies can be performed much more easily with the help of 90°-bent PED tips. With the wide dissection area and the use of straight and 90°-bent PED insert tips (Figure 9), all osteotomies can be easily performed.

The upper lateral incision (Figure 1) we described in the CloPi technique provides direct access to both the ULCs and nasal bones while maintaining a closed approach. This approach also facilitates effective supraperichondrial and subperiosteal dissection in a continuous plane.^13^ Furthermore, preservation of nasal ligaments and soft tissues, as well as reduced edema and ecchymosis, are notable advantages of the CloPi technique.

The aesthetic and functional results of the CloPi procedure were evaluated with the ROE questionnaire and compared with the ROE scores obtained from the control group in which conventional osteotomies were used in closed rhinoplasty. Compared with preoperative values, significant improvements were seen in all categories in both groups, indicating that both techniques were successful both aesthetically and functionally (Supplemental Tables 3, 4). When the improvements in ROE scores were compared between the 2 groups, the CloPi group showed significantly greater postoperative improvement than the control group in all questions except for Question 2, which assessed isolated function. This indicates that the CloPi technique provides significantly better results, particularly in terms of aesthetic outcomes, compared with the conventional technique. Furthermore, although 2 patients in the control group required revision surgery because of dissatisfaction caused by nasal wall and dorsal irregularities, no such complaints were observed in the CloPi group, suggesting that the technique is more satisfactory in terms of aesthetic outcomes. Additionally, no soft tissue damage or burns because of PEDs were observed postoperatively. Overall, all of these findings suggest that the CloPi technique is safe, reliable, and successful with better postoperative outcomes.

The choice between conventional osteotomies and piezoelectric osteotomies during the study period was not randomized. In the early phase of adopting the technique, the PED could not be used in patients with marked axial deviation, a prominent dorsal hump, or an excessively long nose. As surgical experience with the device increased, its applicability broadened to include nearly all primary rhinoplasty cases. Additionally, because the piezoelectric system was rented externally and incurred an additional cost, its advantages and disadvantages were discussed with patients preoperatively, and the technique was not used in cases where the extra expense was declined. Given the retrospective nature of the study, and the use of previously recorded clinical data, the allocation of patients to either technique could not follow a prospective randomized methodology.

This study has several limitations. Its retrospective design and single-surgeon cohort may introduce selection and operator-dependent bias. Aesthetic outcomes were not evaluated with objective or blinded assessment tools, and follow-up durations were not standardized, with some patients having data available for only 3 months. Additionally, the control group had a longer mean follow-up period than the CloPi group, and the more recent adoption and increasing frequency of the CloPi technique may reflect a learning-curve effect that could act as a confounding factor.

## CONCLUSIONS

The CloPi structural rhinoplasty technique described in this study is an approach that allows all necessary osteotomies and nasal tip refinement procedures to be performed safely and effectively in closed rhinoplasty surgeries. The PEDs allowed for controlled bone modifications while minimizing trauma to surrounding tissues and the use of the upper lateral incision improved access and visualization for precise dissection and osteotomies. Through wide surgical dissection, CloPi surgery can be performed easily and, stable and satisfactory results can be obtained.

## Supplemental Material

This article contains supplemental material located online at https://doi.org/10.1093/asjof/ojag021.

## Supplementary Material

ojag021_Supplementary_Data
