# Supplementary material for: Use of Piezoelectric Devices in Closed Structural Rhinoplasty
Source: Aesthet Surg J Open Forum. 2026 Feb 3;8:ojag021. doi: 10.1093/asjof/ojag021 (PMC13098126; doi:10.1093/asjof/ojag021)
Supplement: ojag021_Supplementary_Data [file ojag021_Supplementary_Data.zip › Supplementary Table 2 General Patient of Control.docx]

Table, Supplemental Digital Content 2.: General patient features of control group (closed rhinoplasty technique using conventional osteotomy)

| Patient No. | Age | Gender | Follow-Up Duration (Month) | Revision Surgery |
| --- | --- | --- | --- | --- |
| 1 | 27 | F | 58 | N |
| 2 | 19 | M | 57 | N |
| 3 | 26 | F | 57 | N |
| 4 | 44 | F | 56 | N |
| 5 | 21 | F | 56 | N |
| 6 | 32 | M | 55 | N |
| 7 | 23 | F | 54 | N |
| 8 | 39 | F | 54 | N |
| 9 | 20 | F | 54 | N |
| 10 | 22 | M | 53 | N |
| 11 | 18 | M | 53 | N |
| 12 | 23 | F | 52 | N |
| 13 | 35 | F | 52 | N |
| 14 | 29 | F | 51 | N |
| 15 | 40 | M | 51 | N |
| 16 | 54 | F | 50 | N |
| 17 | 25 | F | 50 | N |
| 18 | 33 | F | 49 | Y |
| 19 | 18 | M | 49 | N |
| 20 | 57 | F | 49 | N |
| 21 | 42 | F | 48 | N |
| 22 | 37 | F | 48 | N |
| 23 | 21 | M | 47 | Y |
| 24 | 59 | M | 46 | N |
| 25 | 46 | F | 46 | Y |
| 26 | 27 | F | 45 | N |
| 27 | 38 | M | 45 | N |
| 28 | 24 | F | 44 | N |
| 29 | 39 | F | 43 | Y |
| 30 | 55 | M | 43 | N |
| 31 | 28 | F | 43 | N |
| 32 | 39 | F | 42 | N |
| 33 | 20 | F | 42 | N |
| 34 | 29 | M | 41 | N |
| 35 | 41 | M | 41 | N |
| 36 | 27 | F | 41 | N |
| 37 | 36 | F | 40 | N |
| 38 | 46 | F | 40 | N |
| 39 | 61 | M | 39 | N |
| 40 | 22 | M | 39 | N |
| 41 | 46 | F | 38 | N |
| 42 | 38 | F | 38 | N |
| 43 | 24 | F | 37 | N |
| 44 | 49 | M | 37 | N |
| 45 | 24 | M | 36 | N |
| 46 | 32 | F | 36 | N |
| 47 | 55 | F | 35 | N |
| 48 | 22 | M | 35 | N |
| 49 | 31 | F | 35 | N |
| 50 | 45 | F | 35 | N |
| 51 | 23 | F | 34 | N |
| 52 | 29 | M | 34 | N |
| 53 | 58 | F | 34 | N |
| 54 | 39 | M | 33 | N |
| 55 | 44 | M | 33 | N |
| 56 | 27 | F | 32 | N |
| 57 | 36 | F | 32 | N |
| 58 | 22 | F | 32 | N |
| 59 | 35 | F | 31 | N |
| 60 | 18 | M | 31 | N |
| 61 | 53 | M | 29 | N |
| 62 | 32 | F | 29 | N |
| 63 | 28 | M | 28 | N |
| 64 | 39 | F | 28 | N |
| 65 | 43 | F | 27 | N |
| 66 | 50 | M | 27 | N |
| 67 | 41 | M | 26 | N |
| 68 | 28 | F | 25 | Y |
| 69 | 35 | F | 25 | N |
| 70 | 47 | F | 25 | N |
| 71 | 33 | M | 24 | Y |
| 72 | 19 | M | 23 | N |
| 73 | 42 | F | 23 | N |
| 74 | 28 | F | 22 | N |
| 75 | 38 | F | 22 | N |
| 76 | 21 | M | 21 | N |
| 77 | 37 | F | 21 | N |
| 78 | 18 | M | 20 | N |
| 79 | 44 | F | 19 | N |
| 80 | 25 | F | 19 | N |
| 81 | 32 | M | 19 | N |
| 82 | 28 | F | 18 | N |
| 83 | 19 | F | 18 | N |
| 84 | 37 | M | 17 | N |
| 85 | 20 | F | 17 | N |
| 86 | 29 | F | 17 | N |
| 87 | 43 | F | 16 | N |
| 88 | 20 | M | 16 | N |
| 89 | 33 | M | 15 | N |
| 90 | 41 | F | 15 | N |
| 91 | 31 | F | 14 | N |
| 92 | 45 | M | 14 | N |
| 93 | 23 | M | 13 | N |
| 94 | 36 | F | 13 | Y |
| 95 | 19 | F | 12 | N |
| 96 | 30 | M | 12 | N |
| 97 | 24 | F | 11 | N |
| 98 | 33 | M | 11 | N |
| 99 | 52 | F | 10 | N |
| 100 | 25 | F | 10 | N |
| 101 | 34 | M | 10 | Y |
| 102 | 23 | F | 9 | N |
| 103 | 48 | F | 9 | N |
| 104 | 18 | F | 9 | N |
| 105 | 29 | M | 8 | N |
| 106 | 25 | F | 8 | N |
| 107 | 51 | M | 8 | N |
| 108 | 39 | F | 8 | N |
| 109 | 29 | M | 7 | N |
| 110 | 47 | F | 7 | N |
| 111 | 35 | F | 6 | N |
| 112 | 21 | M | 6 | N |
| 113 | 33 | F | 6 | Y |
| 114 | 44 | F | 5 | N |
| 115 | 27 | M | 5 | N |
| 116 | 38 | M | 4 | N |
| 117 | 19 | F | 4 | N |
| 118 | 42 | M | 4 | N |
| 119 | 22 | F | 3 | N |
| 120 | 31 | M | 3 | N |

F: Female, M: Male, N: No, Y: Yes
