# Supplementary material for: Use of Piezoelectric Devices in Closed Structural Rhinoplasty
Source: Aesthet Surg J Open Forum. 2026 Feb 3;8:ojag021. doi: 10.1093/asjof/ojag021 (PMC13098126; doi:10.1093/asjof/ojag021)
Supplement: ojag021_Supplementary_Data [file ojag021_Supplementary_Data.zip › Supplementary Table 4 ROE Scores of Control.docx]

Table, Supplemental Digital Content 4: Results of Rhinoplasty Outcomes Evaluation (ROE) questionnaire of control group (closed rhinoplasty technique using conventional osteotomy) ^11^

|  | Q1 | | Q2 | | | Q3 | | | Q4 | | | Q5 | | Q6 | |
| --- | --- | --- | --- | --- | --- | --- | --- | --- | --- | --- | --- | --- | --- | --- | --- |
| Patient | Pre | Post | Pre | Post | Pre | | Post | Pre | | Post | Pre | | Post | Pre | Post |
| 1 | 2 | 3 | 0 | 2 | 3 | | 4 | 2 | | 3 | 2 | | 3 | 1 | 3 |
| 2 | 2 | 4 | 4 | 4 | 2 | | 4 | 3 | | 4 | 2 | | 4 | 1 | 4 |
| 3 | 0 | 2 | 1 | 3 | 1 | | 2 | 1 | | 3 | 1 | | 3 | 1 | 2 |
| 4 | 2 | 3 | 0 | 4 | 2 | | 4 | 2 | | 3 | 1 | | 3 | 0 | 3 |
| 5 | 2 | 4 | 1 | 2 | 2 | | 4 | 2 | | 4 | 2 | | 4 | 2 | 4 |
| 6 | 2 | 3 | 4 | 4 | 2 | | 4 | 2 | | 3 | 2 | | 3 | 1 | 3 |
| 7 | 1 | 3 | 2 | 4 | 1 | | 3 | 1 | | 3 | 1 | | 2 | 1 | 4 |
| 8 | 2 | 3 | 0 | 2 | 2 | | 4 | 2 | | 3 | 2 | | 3 | 2 | 2 |
| 9 | 1 | 4 | 2 | 4 | 2 | | 4 | 1 | | 4 | 2 | | 4 | 1 | 4 |
| 10 | 2 | 2 | 0 | 2 | 2 | | 3 | 2 | | 2 | 2 | | 3 | 1 | 3 |
| 11 | 3 | 3 | 2 | 4 | 3 | | 4 | 3 | | 4 | 3 | | 3 | 2 | 4 |
| 12 | 1 | 3 | 1 | 3 | 1 | | 3 | 1 | | 3 | 1 | | 3 | 1 | 4 |
| 13 | 2 | 3 | 2 | 4 | 2 | | 4 | 2 | | 3 | 2 | | 4 | 2 | 4 |
| 14 | 0 | 4 | 1 | 3 | 1 | | 4 | 0 | | 4 | 1 | | 3 | 1 | 4 |
| 15 | 3 | 3 | 2 | 1 | 3 | | 4 | 3 | | 3 | 3 | | 3 | 3 | 2 |
| 16 | 2 | 3 | 2 | 4 | 2 | | 4 | 2 | | 3 | 2 | | 4 | 2 | 3 |
| 17 | 1 | 3 | 2 | 4 | 2 | | 4 | 1 | | 4 | 1 | | 3 | 2 | 4 |
| 18 | 2 | 1 | 2 | 1 | 3 | | 1 | 2 | | 1 | 3 | | 1 | 2 | 0 |
| 19 | 2 | 4 | 1 | 4 | 3 | | 4 | 2 | | 4 | 2 | | 4 | 1 | 4 |
| 20 | 0 | 3 | 2 | 3 | 0 | | 4 | 0 | | 3 | 0 | | 3 | 0 | 4 |
| 21 | 2 | 4 | 2 | 3 | 2 | | 4 | 3 | | 4 | 2 | | 4 | 2 | 4 |
| 22 | 0 | 4 | 0 | 4 | 0 | | 4 | 0 | | 4 | 0 | | 4 | 0 | 4 |
| 23 | 2 | 3 | 3 | 1 | 2 | | 3 | 2 | | 2 | 2 | | 3 | 2 | 0 |
| 24 | 3 | 4 | 2 | 2 | 4 | | 4 | 3 | | 4 | 3 | | 4 | 3 | 3 |
| 25 | 2 | 2 | 3 | 1 | 3 | | 3 | 2 | | 3 | 2 | | 2 | 2 | 1 |
| 26 | 3 | 4 | 2 | 2 | 4 | | 4 | 3 | | 4 | 3 | | 4 | 2 | 2 |
| 27 | 2 | 4 | 1 | 3 | 2 | | 4 | 3 | | 4 | 2 | | 4 | 2 | 4 |
| 28 | 1 | 2 | 0 | 4 | 2 | | 3 | 1 | | 2 | 2 | | 3 | 1 | 3 |
| 29 | 3 | 2 | 2 | 2 | 3 | | 2 | 4 | | 2 | 3 | | 2 | 2 | 1 |
| 30 | 0 | 2 | 1 | 3 | 1 | | 3 | 1 | | 3 | 0 | | 2 | 0 | 3 |
| 31 | 2 | 4 | 1 | 2 | 3 | | 4 | 2 | | 4 | 3 | | 4 | 2 | 4 |
| 32 | 3 | 4 | 4 | 4 | 4 | | 4 | 3 | | 4 | 3 | | 4 | 3 | 4 |
| 33 | 1 | 4 | 3 | 3 | 2 | | 4 | 2 | | 4 | 1 | | 4 | 1 | 4 |
| 34 | 0 | 2 | 0 | 1 | 0 | | 3 | 0 | | 2 | 1 | | 3 | 0 | 2 |
| 35 | 1 | 1 | 2 | 4 | 1 | | 2 | 2 | | 2 | 1 | | 1 | 1 | 2 |
| 36 | 0 | 4 | 4 | 4 | 0 | | 4 | 1 | | 4 | 1 | | 4 | 0 | 4 |
| 37 | 1 | 2 | 2 | 2 | 1 | | 3 | 2 | | 3 | 2 | | 3 | 1 | 2 |
| 38 | 1 | 2 | 1 | 3 | 2 | | 2 | 2 | | 3 | 1 | | 2 | 1 | 3 |
| 39 | 1 | 1 | 3 | 2 | 1 | | 1 | 1 | | 2 | 2 | | 1 | 1 | 1 |
| 40 | 4 | 4 | 0 | 3 | 4 | | 4 | 4 | | 4 | 4 | | 4 | 2 | 4 |
| 41 | 2 | 2 | 1 | 2 | 2 | | 2 | 2 | | 2 | 2 | | 2 | 2 | 2 |
| 42 | 3 | 2 | 2 | 4 | 3 | | 3 | 3 | | 2 | 3 | | 3 | 3 | 2 |
| 43 | 2 | 3 | 1 | 3 | 2 | | 4 | 1 | | 3 | 2 | | 3 | 2 | 3 |
| 44 | 0 | 2 | 1 | 1 | 0 | | 2 | 1 | | 2 | 1 | | 3 | 0 | 2 |
| 45 | 1 | 2 | 2 | 3 | 1 | | 3 | 1 | | 2 | 1 | | 2 | 1 | 2 |
| 46 | 0 | 4 | 0 | 4 | 0 | | 4 | 0 | | 4 | 0 | | 4 | 0 | 4 |
| 47 | 2 | 3 | 2 | 4 | 2 | | 3 | 2 | | 4 | 2 | | 3 | 2 | 4 |
| 48 | 1 | 2 | 2 | 3 | 1 | | 2 | 1 | | 3 | 1 | | 3 | 1 | 2 |
| 49 | 2 | 2 | 3 | 2 | 2 | | 2 | 3 | | 2 | 2 | | 2 | 3 | 2 |
| 50 | 0 | 2 | 1 | 3 | 0 | | 2 | 0 | | 2 | 1 | | 3 | 0 | 2 |
| 51 | 2 | 1 | 2 | 2 | 2 | | 2 | 2 | | 1 | 3 | | 2 | 2 | 1 |
| 52 | 0 | 3 | 1 | 2 | 0 | | 2 | 1 | | 3 | 0 | | 3 | 0 | 3 |
| 53 | 2 | 3 | 3 | 3 | 1 | | 3 | 2 | | 3 | 2 | | 2 | 2 | 3 |
| 54 | 2 | 3 | 1 | 3 | 2 | | 4 | 2 | | 3 | 3 | | 3 | 2 | 4 |
| 55 | 1 | 2 | 0 | 3 | 1 | | 3 | 1 | | 2 | 1 | | 3 | 1 | 3 |
| 56 | 0 | 3 | 1 | 3 | 1 | | 4 | 1 | | 4 | 0 | | 3 | 0 | 3 |
| 57 | 2 | 3 | 2 | 4 | 2 | | 4 | 2 | | 3 | 3 | | 4 | 2 | 3 |
| 58 | 2 | 4 | 1 | 4 | 2 | | 4 | 2 | | 4 | 2 | | 4 | 2 | 4 |
| 59 | 1 | 1 | 1 | 3 | 1 | | 2 | 1 | | 1 | 2 | | 2 | 1 | 2 |
| 60 | 2 | 3 | 2 | 3 | 2 | | 3 | 3 | | 4 | 2 | | 2 | 2 | 3 |
| 61 | 0 | 3 | 0 | 2 | 0 | | 2 | 0 | | 3 | 1 | | 3 | 0 | 3 |
| 62 | 1 | 3 | 3 | 4 | 2 | | 4 | 1 | | 3 | 1 | | 4 | 1 | 3 |
| 63 | 0 | 2 | 2 | 2 | 1 | | 3 | 0 | | 2 | 0 | | 2 | 1 | 3 |
| 64 | 1 | 3 | 3 | 3 | 1 | | 4 | 1 | | 3 | 1 | | 4 | 1 | 3 |
| 65 | 0 | 2 | 4 | 4 | 0 | | 2 | 0 | | 3 | 0 | | 2 | 0 | 2 |
| 66 | 1 | 2 | 2 | 3 | 1 | | 2 | 1 | | 3 | 1 | | 2 | 1 | 3 |
| 67 | 1 | 3 | 2 | 4 | 1 | | 3 | 1 | | 3 | 1 | | 3 | 1 | 3 |
| 68 | 3 | 2 | 2 | 2 | 3 | | 1 | 3 | | 2 | 3 | | 2 | 3 | 2 |
| 69 | 0 | 4 | 3 | 4 | 0 | | 4 | 1 | | 4 | 0 | | 4 | 0 | 4 |
| 70 | 2 | 3 | 1 | 2 | 2 | | 4 | 2 | | 3 | 2 | | 3 | 2 | 4 |
| 71 | 3 | 1 | 2 | 1 | 3 | | 2 | 4 | | 2 | 3 | | 1 | 3 | 1 |
| 72 | 0 | 2 | 1 | 3 | 0 | | 2 | 1 | | 2 | 0 | | 2 | 0 | 2 |
| 73 | 1 | 2 | 2 | 3 | 1 | | 3 | 1 | | 2 | 1 | | 2 | 1 | 3 |
| 74 | 1 | 3 | 2 | 3 | 1 | | 3 | 1 | | 4 | 1 | | 3 | 1 | 3 |
| 75 | 1 | 2 | 4 | 4 | 1 | | 2 | 1 | | 3 | 2 | | 3 | 1 | 2 |
| 76 | 2 | 2 | 3 | 2 | 3 | | 2 | 2 | | 2 | 2 | | 2 | 2 | 2 |
| 77 | 2 | 3 | 2 | 4 | 2 | | 4 | 2 | | 3 | 2 | | 3 | 2 | 4 |
| 78 | 0 | 4 | 2 | 4 | 0 | | 4 | 0 | | 4 | 0 | | 4 | 0 | 4 |
| 79 | 2 | 3 | 2 | 3 | 2 | | 4 | 2 | | 3 | 2 | | 3 | 2 | 3 |
| 80 | 0 | 2 | 1 | 2 | 1 | | 2 | 0 | | 2 | 0 | | 2 | 0 | 2 |
| 81 | 3 | 2 | 3 | 3 | 3 | | 2 | 3 | | 3 | 3 | | 2 | 3 | 3 |
| 82 | 1 | 3 | 0 | 3 | 1 | | 4 | 1 | | 3 | 1 | | 3 | 1 | 3 |
| 83 | 0 | 2 | 3 | 1 | 0 | | 3 | 0 | | 2 | 0 | | 2 | 0 | 1 |
| 84 | 4 | 4 | 0 | 3 | 4 | | 4 | 3 | | 4 | 4 | | 4 | 4 | 4 |
| 85 | 2 | 4 | 1 | 3 | 3 | | 4 | 2 | | 4 | 2 | | 4 | 2 | 4 |
| 86 | 2 | 2 | 3 | 4 | 2 | | 3 | 2 | | 2 | 2 | | 3 | 2 | 3 |
| 87 | 1 | 4 | 3 | 2 | 1 | | 4 | 2 | | 4 | 2 | | 3 | 1 | 4 |
| 88 | 1 | 3 | 2 | 3 | 1 | | 3 | 1 | | 4 | 1 | | 3 | 1 | 3 |
| 89 | 0 | 2 | 1 | 3 | 1 | | 3 | 1 | | 3 | 0 | | 2 | 1 | 3 |
| 90 | 2 | 3 | 1 | 3 | 2 | | 3 | 2 | | 4 | 2 | | 3 | 2 | 3 |
| 91 | 1 | 3 | 1 | 2 | 1 | | 3 | 2 | | 4 | 1 | | 3 | 1 | 3 |
| 92 | 2 | 2 | 3 | 2 | 2 | | 2 | 2 | | 3 | 2 | | 2 | 2 | 2 |
| 93 | 0 | 3 | 2 | 3 | 1 | | 3 | 0 | | 3 | 0 | | 3 | 0 | 3 |
| 94 | 3 | 1 | 2 | 2 | 3 | | 2 | 3 | | 1 | 3 | | 1 | 3 | 1 |
| 95 | 1 | 2 | 1 | 3 | 1 | | 3 | 1 | | 2 | 2 | | 3 | 1 | 2 |
| 96 | 2 | 4 | 2 | 3 | 2 | | 4 | 1 | | 4 | 2 | | 4 | 2 | 4 |
| 97 | 0 | 4 | 2 | 3 | 0 | | 3 | 0 | | 4 | 0 | | 4 | 0 | 4 |
| 98 | 2 | 3 | 4 | 4 | 2 | | 3 | 2 | | 2 | 2 | | 3 | 2 | 2 |
| 99 | 1 | 3 | 3 | 1 | 1 | | 3 | 1 | | 3 | 1 | | 4 | 1 | 2 |
| 100 | 2 | 3 | 2 | 4 | 2 | | 3 | 2 | | 4 | 2 | | 3 | 2 | 4 |
| 101 | 3 | 0 | 2 | 0 | 3 | | 0 | 3 | | 1 | 3 | | 0 | 3 | 0 |
| 102 | 1 | 4 | 3 | 4 | 1 | | 4 | 0 | | 4 | 0 | | 3 | 0 | 4 |
| 103 | 2 | 2 | 1 | 3 | 2 | | 3 | 2 | | 3 | 2 | | 3 | 2 | 3 |
| 104 | 0 | 3 | 1 | 3 | 0 | | 3 | 0 | | 4 | 1 | | 4 | 0 | 3 |
| 105 | 2 | 4 | 0 | 3 | 2 | | 4 | 3 | | 4 | 2 | | 4 | 1 | 4 |
| 106 | 0 | 2 | 2 | 2 | 0 | | 2 | 1 | | 2 | 0 | | 2 | 0 | 2 |
| 107 | 1 | 3 | 0 | 3 | 1 | | 3 | 1 | | 2 | 1 | | 4 | 1 | 3 |
| 108 | 0 | 4 | 0 | 4 | 0 | | 4 | 0 | | 4 | 0 | | 4 | 0 | 4 |
| 109 | 1 | 3 | 1 | 3 | 1 | | 4 | 1 | | 3 | 1 | | 3 | 1 | 4 |
| 110 | 2 | 3 | 3 | 2 | 2 | | 3 | 2 | | 3 | 3 | | 3 | 2 | 3 |
| 111 | 0 | 2 | 0 | 1 | 0 | | 2 | 0 | | 1 | 0 | | 2 | 0 | 1 |
| 112 | 1 | 3 | 1 | 2 | 1 | | 2 | 1 | | 3 | 1 | | 3 | 1 | 3 |
| 113 | 3 | 2 | 2 | 2 | 3 | | 2 | 3 | | 1 | 3 | | 1 | 3 | 1 |
| 114 | 0 | 3 | 0 | 4 | 0 | | 3 | 0 | | 4 | 0 | | 3 | 0 | 4 |
| 115 | 3 | 4 | 1 | 3 | 4 | | 4 | 3 | | 4 | 3 | | 4 | 2 | 4 |
| 116 | 0 | 2 | 1 | 2 | 1 | | 2 | 0 | | 2 | 0 | | 3 | 0 | 2 |
| 117 | 1 | 3 | 1 | 4 | 2 | | 3 | 1 | | 3 | 1 | | 3 | 1 | 3 |
| 118 | 0 | 2 | 1 | 4 | 1 | | 2 | 0 | | 2 | 0 | | 3 | 0 | 3 |
| 119 | 2 | 3 | 1 | 4 | 2 | | 3 | 2 | | 4 | 2 | | 3 | 1 | 3 |
| 120 | 1 | 3 | 2 | 4 | 1 | | 4 | 1 | | 3 | 1 | | 3 | 1 | 3 |

Q: Question
