# Supplementary figures and images for: Use of Piezoelectric Devices in Closed Structural Rhinoplasty
Source: Aesthet Surg J Open Forum. 2026 Feb 3;8:ojag021. doi: 10.1093/asjof/ojag021 (PMC13098126; doi:10.1093/asjof/ojag021)

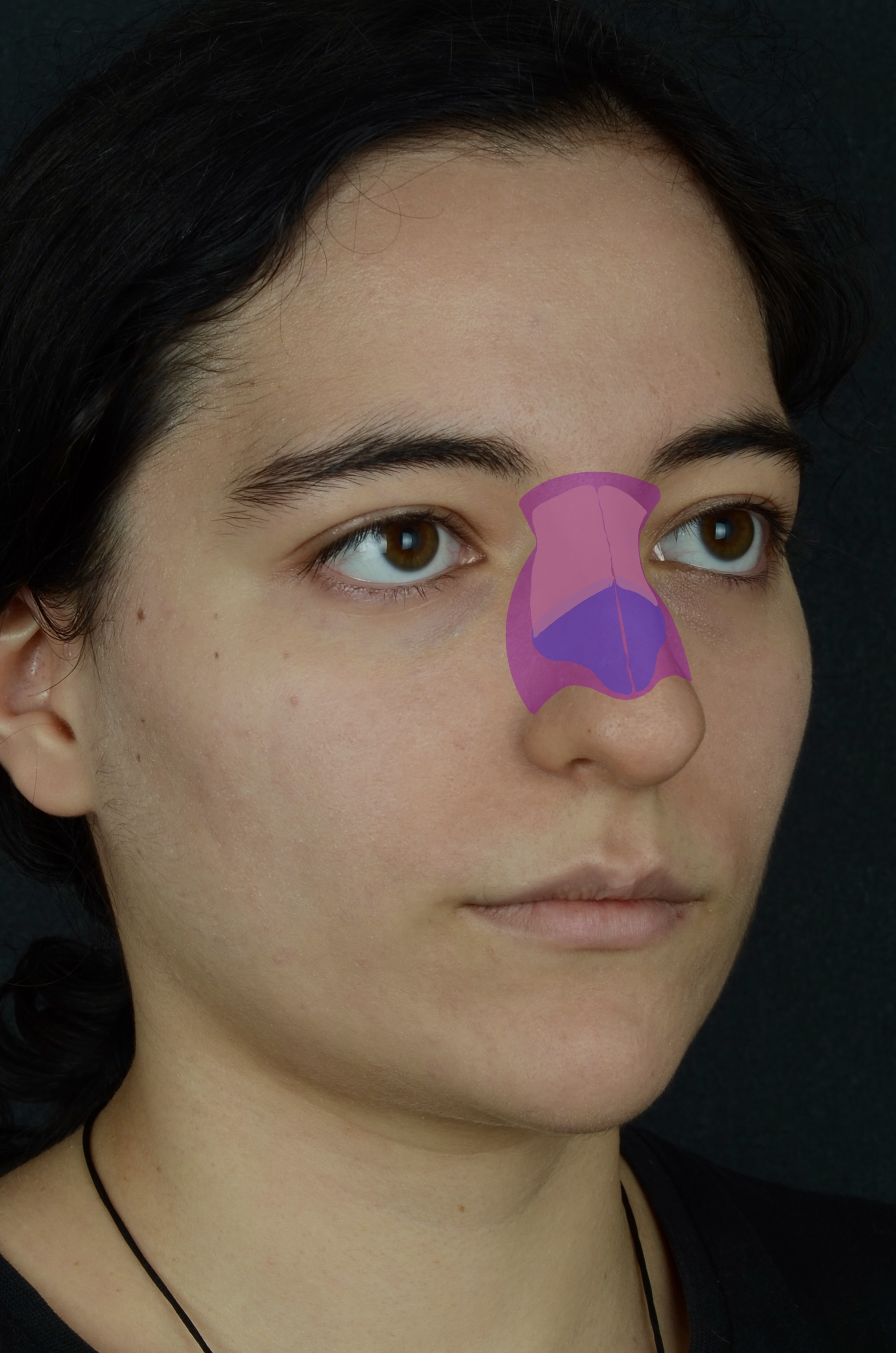

Supplement: ojag021_Supplementary_Data [file ojag021_Supplementary_Data.zip › Supplementary Figure 1.tiff]
